# Do mental health patients learn what their cognitive-behaviour therapists think they do? A short report on qualitative interviews comparing perspectives

**DOI:** 10.1080/17482631.2018.1527598

**Published:** 2018-10-05

**Authors:** Franziska Kühne, Hannah Lesser, Franziska Petri, Florian Weck

**Affiliations:** Department of Psychology, Clinical Psychology and Psychotherapy, University of Potsdam, Potsdam, Germany

**Keywords:** Cognitive-behavioural therapy (CBT), psychotherapy, interview study, learning, skills

## Abstract

**Purpose**: The acquisition of skills is essential to the conceptualization of cognitive-behavioural therapy. Yet, what experiences are encountered and what skills actually learned during therapy, and whether patients and therapists have concurrent views hereof, remains poorly understood.

**Method**: An explorative pilot study with semi-structured, corresponding interview guides was conducted. Pilot data from our outpatient unit were transcribed and content-analyzed following current guidelines.

**Results**: The responses of 18 participants (patients and their psychotherapists) were assigned to six main categories. *Educational and cognitive aspects* were mentioned most frequently and consistently by both groups. Having learned *Behavioural alternatives* attained the second highest agreement between perspectives.

**Conclusions**: Patients and therapists valued CBT as an opportunity to learn new skills, which is an important prerequisite also for the maintenance of therapeutic change. We discuss limitations to generalizability but also theoretical and therapy implications.

## Introduction

Although even in 1961, Bandura stated that it is “customary to conceptualize psychotherapy as a learning process” (p. 143) aiming at behaviour change, there is relatively little research on what skills are addressed by psychotherapy in general and interestingly, by cognitive-behavioural therapy (CBT) in particular (Hundt, Mignogna, Underhill, & Cully, 2013). A narrative review identified preliminary support for skills as mediators between CBT and outcome (Hundt et al., 2013). Yet, they focused on depression only, found that skill use was frequently confounded with outcome assessments, that the efficacy of skill use and its everyday practice were often neglected, and they thus emphasized the need for further psychometric testing of skill measures (Hundt et al., 2013). Methods used for the evaluation of skill use include patient questionnaires, interviews, knowledge tests, thought-listing procedures and daily records with subsequent rater codings, or observational codings of session recordings (Hofmann, Fehlinger, Stenzel, & Rief, 2015; Hundt et al., 2013). Studies have investigated the level of agreement between patients and therapists on outcomes like alliance or functioning (e.g., Bar-Kalifa et al., 2016). Others were conducted with therapists rating their patient’s skill use, which again may introduce bias (Hundt et al., 2013). Nonetheless, the reliable and valid measurement of skills is fundamental to the research of skill use, the differentiation of frequency, quality or efficacy of use, and the comparison of patient and therapist perspectives (Hundt et al., 2013).

As alliance is supposed to reflect the mutual engagement of patients and therapists, its important moderate association with treatment outcome has been replicated repeatedly, it is of relevance for reducing drop-out and pivotal in CBT as in other forms of psychotherapy (Flückiger, Del Re, Wampold, Symonds, & Horvath, 2012; Roos & Werbart, 2013). On the other hand, therapists’ experiences of their work may also predict divergent patient and therapist views of alliance (Hartmann, Joos, Orlinsky, & Zeeck, 2015). Empirical investigations yield information that alliance may facilitate the influence of skills learned during therapy on outcome (Rubel, Rosenbaum, & Lutz, 2017), and agreement between therapists and clients regarding goals and tasks of psychotherapy may be more outcome-relevant than bond (Webb et al., 2011).

Thus, a need persists for empirical studies on the congruence between patients and therapists regarding their perceptions of patient skill use. This is relevant especially to CBT, as it builds on learning principles, is goal- and action-oriented, and focuses on transparency and reproducibility (Gerlach, 2018). For example, psychoeducation, cognitive restructuring or behavioural experiments are assumed to improve patient outcomes, either directly or indirectly, via changes in thoughts, emotions and behaviours.

Based on the current findings, we conducted a pilot interview study to explore what mental health outpatients actually learned during their ongoing CBT, comparing patient and therapist perspectives. We used a qualitative methodology to gain a more profound and comprehensive understanding of skills learned and to generate hypotheses for future studies.

## Method

The current qualitative study is based on individual interviews with mental health patients and their psychotherapists, and analyzed using qualitative content analysis. According to Graneheim, Lindgren, and Lundman (2017), qualitative content analysis describes subjective experiences within certain contexts, and may refer to manifest as well as to latent contents.

### Sampling strategy

Reporting refers to the Standards for Reporting Qualitative Research (O’Brien, Harris, Beckman, Reed, & Cook, 2014; Supplement 1). The study was conducted at our psychotherapeutic outpatient clinic. All adult CBT therapists employed in the clinic at that time (n = 9) were invited to take part, together with one patient who has already had at least eight diagnostic or therapy sessions. All therapists had a Master’s degree in psychology and were licensed CBT therapists. The patients were treated according to the guidelines, for instance, for the treatment of depression or anxiety disorders, published by the Association of the Scientific Medical Societies in Germany. Patients were considered for inclusion by their therapists if they had the appropriate intellectual and language skills for interview participation. One patient dropped out before the interviews due to social anxiety, so that the therapist included another patient. Interviews were conducted concurrently for patients and therapists, and independently by two graduate students (B.Sc. psychology; HL, FP) who were not part of the therapeutic team. Since patients could have felt disturbed by the questions (Lindgren, Sundbaum, Eriksson, & Graneheim, 2014), they were instructed that they should describe as much content as they felt comfortable with, that they could interrupt or terminate participation at each time, they were informed about confidentiality and about the possibility of a subsequent conversation with the first author (licensed psychotherapist) if necessary. Ethical approval was given by the ethics review board of the university (no. 73/2016). Within the accidental sample, all patients gave informed consent and were paid an expense allowance of €20. All were still undergoing psychotherapy approved by their health insurance, i.e., interview participation was independent of psychotherapy allowance and continuance.

### Data collection and analysis

We conducted semi-structured face-to-face interviews from March to May 2017, using corresponding interview guides that asked about learning experiences during the last session and within the current CBT (e.g., Which new experiences did you/your patient have during the current therapy/session? Is there anything new or relevant that you/your patient have/has learned during therapy in general? If yes, what? How do you realize that you/your patient have/has learned something new? etc.). The guides were constructed using funnelling, i.e., starting with broad questions, followed by increasingly concrete ones (Smith, 2007). Mental health issues are often neglected or stigmatized (Lindgren et al., 2014) which required empathic interview conduction. Thus, interview conduction was piloted in 3-hour training. Interviews took 15–40 minutes, were audio-taped, transcribed verbatim, anonymized and deleted no later than 3 weeks after conduction. The interview transcripts were content-analysed inductively (Mayring, 2014) and electronically (MAXQDA, 1989–2017) with the aid of a coding guide with standard examples. First, three transcripts on each perspective were read to familiarize researchers with the data, after which a preliminary category system was derived and discussed so as to foster a common understanding (FK, HL, FP). The main and subordinate categories were derived subsequently by two researchers (HL, FP) and supervised by the corresponding author. Inter-rater agreement (HL, FP) was calculated from three randomly selected patient and therapist interviews each, and was κ = .66-.68 (unmarked transcripts) and κ = .89-.99 (transcripts with marked passages to allocate items to categories) respectively.

## Results

### Sample characteristics

Patients’ mean age was 34 (SD = 15; range 18–61; eight female). Four patients have had prior psychotherapy (2 CBT, 1 CBT and psychodynamic therapy, 1 substance abuse treatment). Patients were diagnosed with depression (n = 5), anxiety (n = 3), somatoform (n = 2), adjustment (n = 1) or personality disorder (n = 1; comorbidities possible). At the time of the interview, they had had an average of 24 therapy sessions (SD = 19; range 9–64). Within the German outpatient setting, with more than 24 sessions, three therapies were within the range of long-term CBT (see Table I). Seven CBT therapists were female with a mean of 8 years (SD = 4; range 5–17) post-graduate experience. All were European.

**Table I.** Patient sample characteristics.

Session number	Previous therapy	Education
9	no	Academic high school
12	no	Academic high school
12	no	Middle school
13	no	Middle school
15	yes (2014, 2010)	Middle school
19	yes (2012)	Academic high school
29	yes (2009, 2015)	Middle school
47	no	University
64	yes (2016, 1997)	Middle school

### Categories with examples of citations

Within *(1) Educational and cognitive aspects*, four subcategories emerged. All patients had *Developed an understanding of their disease*, e.g., psychoeducational knowledge on diagnosis or symptoms. One patient said s/he had learned “*That my very thoughts, in fact it is more about my thoughts […], have a strong impact on my feelings and behaviour” (P5)*.

The importance of *Educational and cognitive aspects* was confirmed by the therapists’ frequently related statements, and as the treating therapist confirmed: *The patient learned the connections between thoughts, feelings and behaviour and how to deal with them […]’ (T5).*

*Having developed more functional cognitions* was another subcategory within the first category. Patients considered the nature of their thoughts more often, learned how to test them and how to develop more functional thoughts. Therapists termed cognitive restructuring as the appropriate CBT term. Most patients also learned to *reflect on themselves* more thoroughly and developed a greater awareness of their own problems: “*At first, I did not consider the actual problems that I had as problems at all, but as something that was just there. […] This has only become clear to me through the therapy” (P9).*

Therapists described the patient’s newly acquired ability to adopt other perspectives and to reflect on their behaviour and actions. Finally, from both perspectives, patients had learned to *recognize patterns in their experiences and behaviour*, and one indicated: “*Well actually, these are always the things that I have tended towards […] that I have taken on too many things that I just could not cope with” (P6).*

Second most often, patients had learned (*2) Behavioural alternatives*. Instead of avoidance, many patients had learned to *cope actively with problems*, for example during exposure to past traumatic experiences: “*[…] What was hidden away for decades or one’s whole life, one has to confront again” (P6).*

Therapists added the importance of patients having learned to discuss unpleasant topics, change habits or reduce avoidance. Furthermore, patients indicated having *extended their behavioural repertoire*: “*I often try to achieve a lot through breathing correctly, which we are practicing again” (P4)*. Accordingly, therapists perceived modified attitudes or skills in their patients: *“S/he had attempted to reactivate inner resources” (T4).*

The category *(3) Self-development* involves having learned to *strengthen one’s self-worth*, as one patient realized: *“That I am not just a useless object, as one so often feels. And that, I believe, is the most important thing I have gained here” (P7).*

From the therapist’s perspective, patients developed confidence and self-acceptance. Additionally, *strengthening self-care*, for instance by taking responsibility for one’s life or by appreciating one’s needs, was an important learning experience for patients, and therapists added patients’ newly developed ability to request for social support.

In terms of *(4) Interpersonal aspects*, both parties indicated patients having learned to *strengthen their ability to distance themselves from problem areas*, and one patient expressed it as follows: *“Precisely because this acute situation had arisen again, today, here was the awareness: Distance, distance and fixed rules” (P2).*

Furthermore, patients perceived the need to *deal differently with conflicts*, by reacting less aggressively or standing up for one’s opinions. Instead of conflict management, therapists added *enhanced communication skills* for their patients.

Regarding category *(5) Emotion regulation*, from both perspectives, patients had learned how to *modulate their tension* (such as via relaxation techniques) and how to *deal differently with strong emotions*.

Within *6) Acceptance*, patients and therapists stated that the former had *developed a realistic perspective regarding changes* (e.g., that changes need time). Patients added they were now able to *accept and sustain difficulties* (e.g., accept what you cannot explain).

### Congruence between patient and therapist perspectives

**Figure 1.**
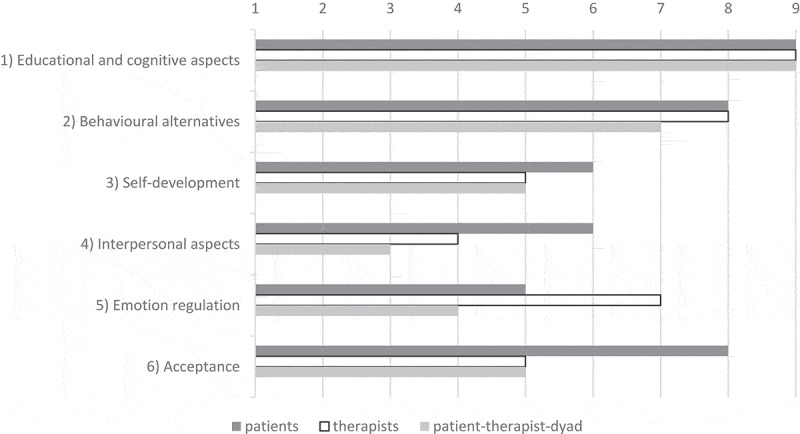
Number of *patients*/*therapists* making at least one statement within each category; and number of *patient–therapist dyads* with congruent statements.

Below, we report the total number of indications per category. Most frequently and consistently, (*1) Educational and cognitive aspects* were mentioned by patients (n_pat_ = 62) and therapists (n_th_ = 47) alike (see Figure 1). Four further main categories, having learned (*2) Behavioural alternatives* (n_pat_ = 43, n_th_ = 28), (*3) Self-development* (n_pat_ = 24, n_th_ = 26), (*4) Interpersonal aspects* (n_pat_ = 20, n_th_ = 22) and (*5) Emotion regulation* (n_pat_ = 12, n_th_ = 16) were derived, with *Behavioural alternatives* yielding the second highest agreement between perspectives. The last category, (*6) Acceptance*, was mentioned least often (n_pat_ = 8, n_th_ = 12), and agreement was moderate (Figure 1). Patients used the interviews to reflect on their therapies, and some decided to subsequently feed their experiences back to their therapists.

## Discussion

We explored the skills that patients had learned during their ongoing CBT via face-to-face interviews, and compared patient and therapist perspectives in one of the first pilot studies in this context. Accordingly, we focused not only on depression (Barnes et al., 2013; French et al., 2017; Hundt et al., 2013) but included different diagnoses, which also implied heterogeneity. Study limitations refer to the small accidental sample size, reactivity due to an awareness of being interviewed, self-assessment bias (Walfish, McAlister, O’Donnell, & Lambert, 2012), patient selection by therapists (which is why it remains unclear whether prototypical or especially cooperative patients were included), of therapies mainly in the early stages, and the restriction to a university treatment setting. In future studies, learning and skills use could be addressed routinely and repeatedly as a complete survey within a certain time period, both to reduce bias, and to underscore the importance of skills use.

We chose an explicit and reproducible framework, thus presuppositions and psychological experience were noticed and regarded during interview conduct, analysis and interpretation (Barnicot, Couldrey, Sandhu, & Priebe, 2015; Supplement 2). In line with the qualitative-quantitative approach of content-analysis (Mayring, 2014), code saturation was achieved after the seventh patient and sixth therapist interview, i.e., no new categories emerged, which supports the category system derived. Whereas sample size is not a quality criterion in qualitative research *per se*, rather both, saturation and inter-rater agreement support the trustworthiness of our analyses. Even so, due to the small sample size, we did not evaluate meaning saturation, which, in order to gain a richer understanding of the topic (Hennink, Kaiser, & Marconi, 2017), constitutes a major task for a subsequent, larger study.

In line with other research, patients valued therapy as an opportunity to learn new and diverse cognitive-behavioural skills (Barnes et al., 2013; French et al., 2017), which seems to be an important mediator of symptom change (Hundt et al., 2013). Patient and therapist perceptions strongly relate to general mechanisms of psychotherapeutic change, like resource activation or mastery (Gassmann & Grawe, 2006). Nonetheless, patient–therapist agreement was higher on cognitive and behavioural aspects than on interpersonal aspects or acceptance (emphasized more by patients) or emotion regulation (emphasized more by therapists). Whereas cognitive and behavioural aspects are apparently associated with CBT, self-development and interpersonal aspects relate more to transdiagnostic mechanisms. This is also the case for emotion regulation and acceptance, whereas both are described as part of the so-called “third wave” interventions. Further studies should focus on facilitators of change regarding current daily behaviours and on enablers of long-term change (Barnes et al., 2013; Hundt et al., 2013). Alongside focusing on the benefits of CBT, patients should also be given balanced information about potential obstacles, like emotional problems arising during homework tasks, in order to manage expectations, prepare patients for coping with difficulties, and thus reduce treatment discontinuation (Barnes et al., 2013). Most importantly, patients who valued the learning experiences during CBT are more likely to use newly acquired skills, even in the long run (French et al., 2017).

## Conclusion

Our qualitative pilot study indicates agreement among mental health patients and their therapists regarding cognitive-behavioural skills learned during therapy, which is important for therapeutic change and the maintenance of effects. On the other side, lower congruence on interpersonal factors, acceptance and emotion regulation highlights that divergent priorities were set or language used by patients and therapists. Further research could focus on these divergences and their influence on therapy processes and outcomes.

## Data Availability

German interview guides, the coding guide and the category system are available upon request from the corresponding author.
